# The Impact of Environmental Conditions on Urban Eco-Sustainable Total Factor Productivity: A Case Study of 21 Cities in Guangdong Province, China

**DOI:** 10.3390/ijerph17041329

**Published:** 2020-02-19

**Authors:** Haidong Yu, Juanjuan Zhao

**Affiliations:** 1 School of Economics, Sichuan University, Chengdu 610065, China; 2017321010020@stu.scu.edu.cn; 2 College of Horticulture and Landscape Architecture, Southwest University, Chongqing 400715, China

**Keywords:** ecological index (EI), urban resource consumption index (URCI), urban pollution discharge index (UPDI), ecological sustainable total factor productivity (ESTFP), DEA-Malmquist index, system GMM model

## Abstract

Environmental protection has attracted much attention. This study first describes the status of the ecological environment and then uses data envelopment analysis and the system the system generalized method of moments (GMM) model to study the relationship between the environmental status and ecological sustainable total factor productivity (ESTFP) in 21 prefecture-level cities of Guangdong Province. The main conclusions of this study are as follows. (1) The ecological index (EI), which reflects the ecological environment, shows a general trend of first decreasing and then rising. The average EI value decreased from 80.95 in 2008 to 68.71 in 2011 and then gradually increased to 74.76 in 2017. (2) The ecological sustainable total factor productivity (ESTFP = 0.960), including the two additional dimensions of the urban resource consumption index (URCI) and urban pollution discharge index (UPDI), is better than the traditional total factor productivity (TFP = 0.954). (3) The EI has a highly significant positive promoting effect on ESTFP at a significance level of 1%. The methods and results from this research provide an important scientific reference for the research on urban production efficiency and sustainable urban development in China.

## 1. Introduction

After 40 years of rapid economic development, China’s economy has entered a new normal stage. At present, China’s urbanization construction is in full swing, and it is becoming a new engine to promote economic development. It is undeniable that in the past 40 years of reform and opening up, and in the early stage of urbanization, many local governments, due to their lack of understanding of environmental issues, have blindly taken economic development as the most important development goal. Most of the keywords they put forward are “development” and “utilization”. The total economic volume has indeed increased, but there is serious environmental pollution, ecological resources depletion, and other negative phenomena everywhere. Based on the government’s national policy, in recent years, “sustainable”, “ecological”, and “restoration” have become hot words, which indicates that the government and the public have begun to realize that urban development should be based on sustainable ecological development.

The assessment of the ecological environment involves a study of the current situation and examines changing the rules of the ecological environment. The establishment of an assessment index system and the selection of appropriate assessment methods to assess the environment provides the basis for the protection and restoration of the ecological system. At the same time, the assessment of the ecological environment provides an important means to coordinate the relationship between the regional economy and environmental protection, and to realize regional sustainable development.

With the passage of time, the world’s ecological protection and restoration is imminent. More and more attention has been paid to the application of ecological environment assessment. Niemi and McDonald [1] summarized the wide application of ecological environment assessment indicators in the field of science. Heink and Kowarik [2] gave a general definition of the different meanings of indicators in ecological and environmental planning and put forward suggestions for their use. Brazner et al. [3] showed a method to assess the impact of geographic, geomorphic, and human interference on ecosystem indicators across a wide space scale, and they studied how the ecological indicators of human interference change on the actual wide space scale. The above is the research of non-Chinese scholars on ecological environment assessment; it has mainly involved index design, index definition, and applicable objects. These scholars pointed out that the ecological environment assessment indicators have wide appeal to scientists, environmental managers, and the public. People have been using ecological environment assessment indicators to detect the changes in nature, to evaluate the environmental situation, and to act as a warning signal of ecological problems and a barometer of the trend of ecological resources. However, due to the different emphases and national conditions, most of the research contents are based on the ecological environment of each researcher’s own country. With regard to China, the Ministry of Environmental Protection of China has issued the official assessment system of the Technical Criterion for Ecosystem Status Evaluation (Trial) (HJ192–2015), which was first launched by the Ministry of Environmental Protection in 2006 and adjusted in 2015, thereby unifying the standards for assessment of the national ecological environment. This evaluation system has also become the foundation for the evaluation of the ecological environment in industry. Some Chinese scholars have also conducted relevant research [4,5,6] on all parts of China with the help of this official evaluation system. According to the summary of these studies in China, most scholars used the evaluation system issued by the government to measure the data and evaluate the ecological environment of each region. However, there has been little research on the relationship between the evaluation system and regional economic efficiency. However, these studies only focus on the environmental assessment of a specific place. Compared with the environmental assessment, the research on environmental efficiency, such as green total factor productivity (GTFP) [7,8] and ecological efficiency (EE) [9], is richer. These studies mainly involve concept definition [10,11], index design, and measurement. However, these studies have a long history, and their methods are now a little outdated, thus, they cannot decompose the efficiency to carry out detailed research. An example of this is the World Business Council for Sustainable Development (WBCSD) constructed the measurement index of ecological efficiency at the 1992 Rio Earth Summit: ecological efficiency = product and service value/ecological environment load. Since then, the evaluation of ecological efficiency has changed from qualitative research to quantitative research. Although the ecological efficiency can be measured, the data obtained are a comprehensive total efficiency, which cannot be decomposed, thus, we do not know whether any changes revealed are because of the impact of technological improvement on ecological efficiency or because of the change of pure technological efficiency and scale efficiency. With regard to the “ecological environment load”, Muller and Sturm [12] and UNCTAD [13] thought that it includes two parts: “resource consumption” and “pollution emissions”. Seppalaa et al. [14] and Zhang et al. [15] thought that resource consumption can be considered as the consumption of various ecological resources. Pollution discharge is the discharge of various wastes and pollutants, and these mainly include the discharge of wastewater, waste gas, and other wastes. This provides a large framework for follow-up research. Under the framework of TFP, many scholars expanded their research on TFP by adding factors such as energy consumption and the environment [16,17]. However, because the input and output indicators are not the same, it cannot well represent the ecological sustainable growth. In terms of output, these studies regard GDP as the expected output and environmental pollution as the unexpected output. Energy consumption, capital, and labor are taken as the investment. There is no doubt that this research framework is completely correct, but there are also defects in the following aspects. First, in respect of the unexpected output, there is no unified standard for pollution emission indicators in the unexpected output, such as NO_2_, PM_10_, CO_2_, SO_2_, and COD, which are pollution emissions, but many scholars have not fully included them. Only one or several indicators are considered, which is not comprehensive [18]. Second, in terms of investment, only energy consumption as the consumption of the ecological environment is included [19,20]. The whole of the ecological resources, such as water, land, forest, and other resources, should be taken into account. Third, because the input and output indicators are not the same, the final results will be different, or the conclusions may even be completely opposite.

TFP has been studied for decades in China and elsewhere, and TFP is the main research direction in the economic field. TFP was first proposed by Solow [21]; it is also known as the Solow residual. It mainly studies economic growth through the input (capital and labor) and output (GDP). It refers to the progress of an economy’s own strength after excluding the production factors such as capital and labor. TFP is widely used in industry, agriculture, and finance [22]. With regard to the measurement method of TFP, there are roughly three types of measurement methods: the growth accounting method, Solow production function method, and production frontier method. The growth accounting method [23,24,25] usually calculates the share of the labor force and capital first and then the share of total factor productivity. The key point is to estimate the share of the capital and labor factors. However, this method does not set the production function, it implies that capital and labor can be completely substituted, and the marginal productivity is constant, which is unreasonable. The basic idea of Solow’s production function method [26,27,28] is as follows. First, estimate the total production function, second, calculate the output growth rate of the function, third, deduct the growth rate of the input factors with the output growth rate, thereby obtaining the residual, and fourth, calculate the TFP growth with the residual. This method is also called the production function method and it commonly uses the Cobb-Douglas function. Under the assumption of constant scale returns and Hicks neutral technological progress, TFP growth is equal to the rate of technological progress. The method is based on the neoclassical growth theory; it has few considerations and simple estimation, but its main disadvantage is that there are many assumptions.

In contrast, the production frontier method assumes that if the economic resources are fully utilized, the growth of TFP is equal to the rate of technological progress. One of the characteristics of this method is that it can effectively decompose the growth of TFP, but this method is only applicable to panel data. At present, data envelopment analysis (DEA), which is commonly used [22,29,30] in nonparametric analysis, is classified as a production frontier analysis method. This method can directly use linear optimization to estimate the boundary function and distance function without making assumptions on the function form and distribution. Based on the above analysis and the actual situation, this study uses DEA to estimate the change of the ecological sustainable total factor productivity.

Because of the rising awareness of environmental protection, on the one hand, governments at all levels should pay attention to economic development, but on the other hand, they should protect the environment. In the dilemma between economic development and environmental protection, only considering one of these aspects will not help to solve the problem, thus, the question is, how can the economy be developed on the basis of environmental protection? How can the relationship between environmental protection and economic development be coordinated? How can environmental factors be integrated into the research framework of economic efficiency? Is the economic efficiency, including environmental factors increasing or decreasing? These problems need to be considered from the perspective of economic and environmental coordination. The goal of this research is to study the environmental conditions of Chinese cities from point to area and measure the economic efficiency of the cities according to the evaluation of their environmental conditions. This approach will provide references to assist the relevant governments and decision makers to improve the urban environment and increase the economic efficiency.

Based on this, the potential innovations of this study are as follows. First, this study proposes a new indicator “ecological sustainable total factor productivity (ESTFP)”. The two dimensions of the urban resource consumption index (URCI) and urban pollution discharge index (UPDI) are added to the traditional TFP framework, which can better measure the economic development of a city while taking into consideration the ecological sustainability. Compared with previous studies under the framework of traditional TFP, taking into consideration the consumption of water, electricity, liquefied petroleum gas, land, as well as the emission of waste gas, wastewater, smoke dust, and solid waste, can more comprehensively reflect the sustainable economic growth. Second, we use the DEA Malmquist method to measure the changes and components of ESTFP in 21 prefecture-level cities of Guangdong Province from 2009 to 2016. Third, an empirical study is conducted on the relationship between the ecological environment and ESTFP. 

**Figure 1:**
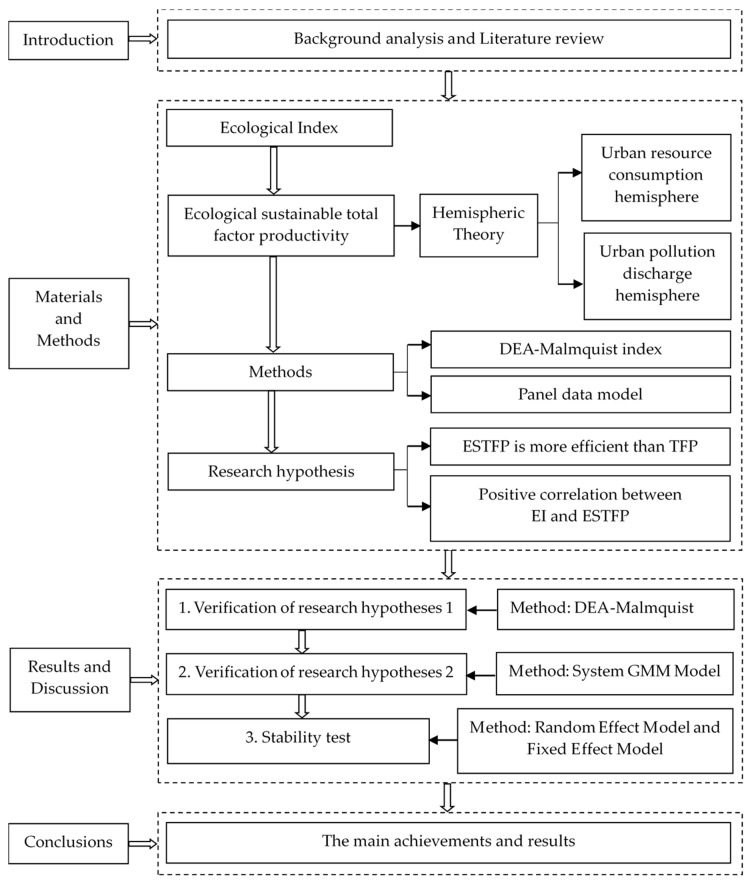
Research structure.

The structure of this paper is as follows (see Figure 1). The second section introduces the related concepts and calculation formulas of ESTFP, URCI, and UPDI and puts forward the research hypothesis and relevant empirical methods (data envelopment analysis and the system GMM model). The third section presents the results and discussion. In summary, we first calculate the ESTFP, we then carry out empirical research on the relationship between the ecological environment and ESTFP, and finally, we discuss the empirical results. The fourth section provides the conclusions. 

## 2. Materials and Methods 

### 2.1. Ecological Index 

With regard to the evaluation of the urban ecological environment, the Technical Criterion for Ecosystem Status Evaluation (Trial) (HJ192–2015) issued by the Ministry of Environmental Protection is the only official standard in China at present. According to the technical criterion, the significant factors that are dominant in the process of ecological damage and protection include the biological richness index, vegetation coverage index, water network denseness index, land stress index, pollution load index, and environmental restriction index. If these factors are combined with a certain weight, the ecological index can be calculated (see Formula (1)). The urban ecological index is used to evaluate the ecological environment quality of cities or urban agglomerations; it has a value range of 0–100. The calculation equation is as follows (see Equation (1)), please see the Appendix A for more details.

Ecological index = 0.35 × Biological richness index + 0.25 × Vegetation coverage index + 0.15 × Water network denseness index + 0.15 × (100-Land stress index) + 0.1 × (100-Pollution load index) + Environmental restriction index (1)

According to the ecological index, the ecological environment is divided into five levels: excellent (EI ≥ 75), good (55 ≤ EI < 75), general (35 ≤ EI < 55), poor (20 ≤ EI < 35), and poorer (EI < 20). According to Formula (1), we can get the EI data of 21 prefecture-level cities in Guangdong Province, as shown in Table 1.

**Figure 2:**
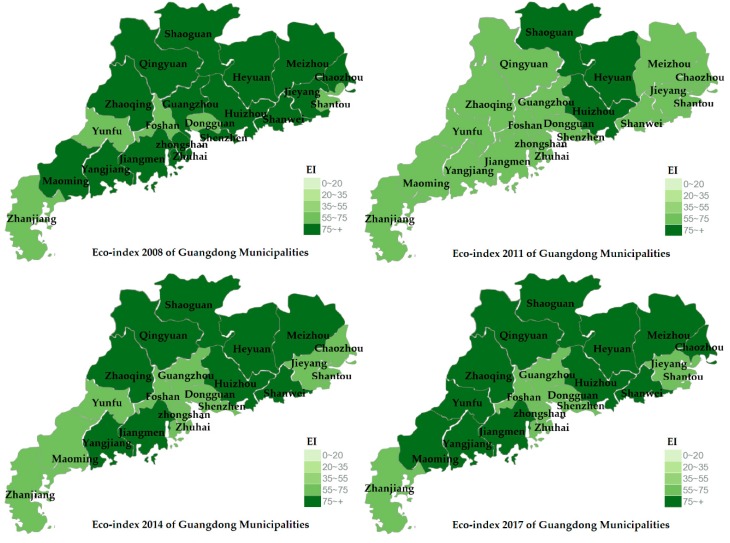
Ecological environment in Guangdong Province.

Using the data in Table 1, this study draws the ecological environment map for some years (Figure 2). From Table 1 and Figure 2, it can be seen that the overall ecological environment of the 21 prefecture-level cities in Guangdong Province shows a trend of first declining and then rising: that is, the dynamic change process of the ecological environment goes from good to bad and then from bad to better. In the existing data, 21 prefecture-level cities are rated as “excellent” and “good”, which indicates that the vegetation coverage is high, the biodiversity is rich, and the ecosystem is stable, thus, they are more suitable for human survival. Among them, the best ecological environment is in 2008, where 16 cities are in the “excellent” category, which indicates that the ecological environment of Guangdong Province is very suitable for human living. The worst ecological environment is in 2011, in which only three cities (Shaoguan, Heyuan, and Huizhou) are at the “excellent” level, and 18 cities are at the “good” level. The ecological environment deteriorated year by year between 2008 and 2011, and the number of “excellent” cities rapidly decreased from 16 in 2008 to 3 in 2011. The ecological environment improved year by year between 2011 and 2017, and the number of “excellent” cities increased year by year from 3 in 2011 to 12 in 2017. The dynamic change process of the ecological environment in 21 prefecture level cities in Guangdong Province is from good to bad then from bad to good. The main reason is that in the early years, governments at all levels and the public did not pay attention to environmental protection, and their economic development was at the expense of the ecological environment. In recent years, with the strengthening of environmental protection by the government, the ecological environment has been improved year by year, and the economy has also developed well. China also benefits from the fact that it is a centralized country. It is very easy to carry out laws and regulations on environmental protection in a centralized country. Its main feature is that it can concentrate on major issues and effectively organize human, material, and financial resources to implement environmental protection measures.

### 2.2. Ecological Sustainable Total Factor Productivity

#### 2.2.1. ESTFP Calculation Framework and Urban Ecological Sustainable Hemispheric Theory

**Figure 3:**
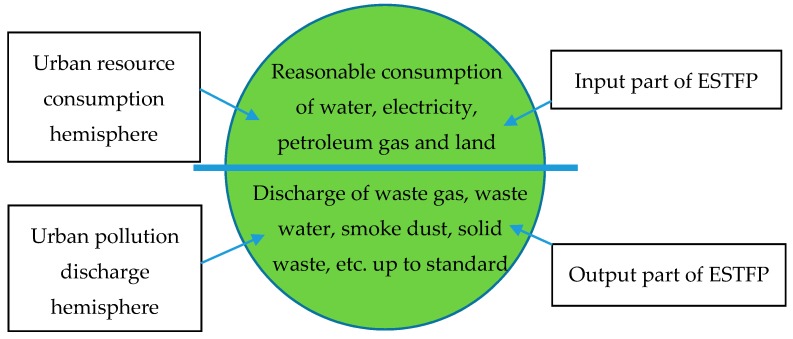
Urban Ecological Sustainable Hemisphere.

In recent years the extensive economic growth mode has brought rapid economic growth with a consequent GDP advantage. This has inevitably been accompanied by a large amount of resource consumption and environmental pollution, which seriously affects and restricts the sustainable development of the future economy. This kind of disregard for a lot of damage to the environment in order to generate high-speed economic growth is also due to the neglect of the resources and environmental constraints in the evaluation system. The traditional TFP only considers the input part of capital and labor, as well as the output part of the total economic volume, and the factors of resource consumption and pollution emission closely related to sustainable development are often ignored. Neglecting the efficiency measurement of resource consumption and pollution emission is bound to be biased. It is difficult to accurately measure the level of urban development from the calculated TFP, and this may bring inappropriate policy recommendations. Based on the reality of the ecological environment, under the framework of traditional TFP, this study integrates two comprehensive indexes into the traditional TFP framework to measure the sustainable growth. According to the definitions of the United Nations Development Program [31], one is the urban resource consumption index (URCI), which reflects the consumption of urban ecological resources, and the other is the urban pollution discharge index (UPDI), which measures the emission dimension of urban pollutants. In this way the estimated ESTFP can effectively measure the economic growth of a city while taking into consideration its ecological sustainable development. Based on the TFP framework and the idea of the urban development hemispheric theory proposed by the United Nations Development Program [31], this study proposes the “urban ecological sustainable hemispheric theory”. See Figure 3.

We divide urban ecological sustainability into two hemispheres. The upper hemisphere is called the “urban resource consumption hemisphere”, which is one of the input parts of ESTFP; it mainly includes the consumption of water, electricity, oil, gas, and land. The lower hemisphere is called the “urban pollution discharge hemisphere”, which is one of the output parts of ESTFP; it mainly includes the discharge of urban waste gas, wastewater, smoke dust, and solid waste. The matching degree of the two hemispheres reflects the level of urban ecological sustainable development. No matter which city, in the process of economic development, it must be clear that the capacity of the ecological resources and ecological environment is limited.

**Figure 4:**
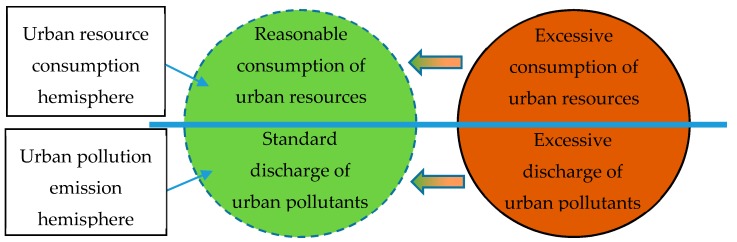
Matching of the Urban Ecological Sustainable Hemispheres.

In the actual situation, there are three kinds of dislocation between the hemispheres of urban resource consumption and urban pollution discharge. First, the ecological resources consumed by urban development exceed their supply capacity, and excessive consumption occurs in the urban resource consumption hemisphere. Second, the urban pollutant emissions are increasing. They exceed the self-cleaning ability of the ecological environment, and excessive emissions appear in the urban pollutant emissions hemisphere. Third, in the process of urban development, excessive consumption of urban resources and excessive emissions of urban pollution occur simultaneously. In Figure 4 the two dashed hemispheres represent a high degree of matching, which is the ideal state for urban ecological sustainable development. The two solid line hemispheres represent the reality; that is, the reality of the destruction of the ecological environment. In the process of ecological sustainable development, cities should use various ways to make their two hemispheres develop in the direction of the arrow so that the two hemispheres can reach the matching state again. 

#### 2.2.2. Input-Output Variable Selection and Data Sources

Input index: (1) Capital input (k). In this study, capital stock is used to replace capital investment, and the method of Zhang [32] is used to estimate the material capital stock using the perpetual inventory method. (2) Labor input (L). In this study, the total number of employees at the end of the year is used as the labor input. (3) Urban resource consumption index (URCI). The index consists of four sub-indexes: the urban water consumption index (UWCI), which is measured by the per capita annual water supply, the urban land consumption index (ULCI), which is measured by the per capita built-up area divided by the permanent population, the urban LPG consumption index (ULPGCI), which is measured by the per capita LPG supply, and the urban electric power consumption index (UEPCI), which is measured by the per capita electricity consumption. The above four sub-indexes are divided by the total number of permanent residents in each city to get the per capita indicators, and then the urban resource consumption index (URCI) is constructed after taking the average value of the calculation through dimensionless processing. All the above aggregate data are from the Guangdong statistical yearbook.

(2) $$\mathrm{URCI}=\frac{\left(\mathrm{UWCI}+\mathrm{ULCI}+\mathrm{ULPGCI}+\;\mathrm{UEPCI}\right)}{4}$$

Output index: (1) The GDP value is selected as the output in this study. Because of inflation, the GDP published every year is the nominal GDP. This study uses the GDP deflator to convert the nominal GDP into the real GDP output value based on 2008. (2) The urban pollution discharge index (UPDI), which consists of four sub-indexes. They are measured, respectively, by the urban water pollution discharge index (UWPDI), which is measured by the total amount of urban wastewater discharge, the urban air pollution discharge index (UAPDI), which is measured by the total amount of urban industrial exhaust discharges, the urban smoke and dust discharge index (USDDI), which is measured by the industrial dust discharge, and the urban solid waste discharge index (USWDI), which is measured by the amount of industrial solid waste. The above four sub-indexes are divided by the total number of permanent residents in each city to get the per capita indicators, and then the urban pollution discharge index (UPDI) is constructed after taking the average value of the calculation through dimensionless processing. All the above aggregate data are from the Guangdong Statistical Yearbook.

(3) $$\mathrm{UPDI}=\frac{\left(\mathrm{UWPDI}+\mathrm{UAPDI}+\mathrm{USDDI}+\;\mathrm{USWDI}\right)}{4}$$

To select the environmental pollution indicators, most researchers use a single indicator to measure. For example, Shi and Li [33] only use CO_2_, and Zhang and Tan [34] only use SO_2_ as the unexpected output. It is difficult to use a single indicator to measure the actual level of environmental pollution in a certain area. It is necessary to consider all kinds of environmental pollution indexes in the same framework depending on the data availability, thus, this study uses the urban pollution discharge index (UPDI) to measure the urban pollutant discharge.

### 2.3. Methods

#### 2.3.1. DEA-Malmquist Index

This section introduces a DEA index method to estimate the change of ESTFP and its decomposition. The Malmquist index was first proposed by Sten Malmquist [35], a Swedish economist, in 1953 to study the changes of consumption in different periods. Caves et al. [36] began to apply this index to the measurement of production efficiency changes. Färe et al. [29] combined a non-parametric linear programming method of this theory with the data envelopment analysis (DEA). Using the method of Färe et al. [29], the production possibility set S^t^ is expressed as follows:

(4) $$s^{t}=\left\{\left(x^{t},y^{t}\right):x^{t}{\mathrm{can}\;\mathrm{produce}\;y}^{t}\right\}$$

where x denotes the inputs, which include the labor force, capital stock, and URCI, and y denotes the output, which includes the GDP and UPDI. First, we have calculated the distance function by DEA, and this was followed by the estimation and reduction of the Malmquist index by the distance function. The distance function can compare the multi-input and multi-output production techniques under unconstrained conditions, analyze the changes caused by the small changes of the input vectors under a given output vector, and use the input distance function to characterize the technical features. The Malmquist productivity index is based on the benchmark technology. The Malmquist indices of T_t_ and T_t+1_ reference technology are calculated as:

(5) $$M^{t}\left(x^{t+1},y^{t+1},x^{t},y^{t}\right)=\frac{D^{t}\left(x^{t+1},y^{t+1}\right)}{D^{t}\left(x^{t},y^{t}\right)}$$

(6) $$M^{t+1}\left(x^{t+1},y^{t+1},x^{t},y^{t}\right)=\frac{D^{t+1}\left(x^{t+1},y^{t+1}\right)}{D^{t+1}\left(x^{t},y^{t}\right)}$$

where $M^{t}$ and $M^{t+1}$ are the Malmquist index of T_t_ and T_t+1_ periods, $\left(x^{t+1},y^{t+1}\right)\;\mathrm{and}\;\left(x^{t},y^{t}\right)$ represent the input and output vectors of the T_t+1_ and T_t_ periods, respectively, and $D^{t}\left(x^{t+1},y^{t+1}\right){\;\mathrm{and}\;D}^{t}\left(x^{t},y^{t}\right)$ represent the distance functions of the ecological sustainable development at time T_t_ and T_t+1_, respectively, with respect to the frontier production technology at time T_t_. Similarly,${\;D}^{t+1}\left(x^{t+1},y^{t+1}\right){\;\mathrm{and}\;D}^{t+1}\left(x^{t},y^{t}\right)$ are the distance functions of ecological sustainable development at time T_t_ and T_t+1_, respectively, with respect to the frontier production technology at time T_t+1_.

When it is combined with the previous four distance functions, the Malmquist index of the productivity change from T_t_ to T_t+1_ can be further obtained as follows:

(7) $$M^{t}\left(x^{t+1},y^{t+1},x^{t},y^{t}\right)={\left[\frac{D^{t+1}\left(x^{t+1},y^{t+1}\right)}{D^{t+1}\left(x^{t},y^{t}\right)}\times \frac{D^{t}\left(x^{t+1},y^{t+1}\right)}{D^{t}\left(x^{t},y^{t}\right)}\right]}^{1/2}$$

Further decomposition can be obtained as:

(8) $$\begin{array}{ll} M^{t}\left(x^{t+1},y^{t+1},x^{t},y^{t}\right) & =\frac{D^{t+1}\left(x^{t+1},y^{t+1}\right)}{D^{t}\left(x^{t},y^{t}\right)}\times {\left[\frac{D^{t}\left(x^{t+1},y^{t+1}\right)}{D^{t+1}\left(x^{t+1},y^{t+1}\right)}\times \frac{D^{t}\left(x^{t},y^{t}\right)}{D^{t+1}\left(x^{t},y^{t}\right)}\right]}^{1/2}\\ & =\mathrm{ESEC}\times \mathrm{ESTC}\\ & =\left(\mathrm{ESPEC}\times \mathrm{ESSEC}\right)\times \mathrm{ESTC} \end{array}$$

In Equation (8), the first part is the ecological sustainable efficiency change (ESEC) and the second part is the ecological sustainable technical change (ESTC). ESEC can be further decomposed into the ecological sustainable pure efficiency change (ESPEC) and the ecological sustainable scale efficiency change (ESSEC).

#### 2.3.2. Panel Data Model

The system GMM model can find out the influence of the explanatory variables on the explained variable, and it can also determine whether the explained variable itself has an impact on them. In terms of the research methods, the static panel is based on the fact that there is no lag effect in the production efficiency, but in fact, everything has a continuous development process; that is, the former period will affect the later period. This study examines the method of building a dynamic panel by the System GMM model. 

In the dynamic panel model, the lag term of the explained variable is introduced into the regression model as the explanatory variable, which gives the model a dynamic interpretation ability, but there are endogenous problems in the model. In order to solve this endogeneity, Arellano and Bond [37] proposed the generalized method of moments (GMM), which uses instrumental variables to derive the corresponding moment conditions; this is the so-called “differential GMM” method. There is a serious “weak instrumental variable” problem in this method, and this leads to the poor accuracy of the coefficient estimation results. Arellano and Blundell [38] proposed a solution to this problem. Based on the new composite moment condition, the system GMM method was proposed. This method combines the difference GMM with the level GMM and takes the difference equation and the level equation as an equation system to estimate the GMM; this is called the system GMM. The system GMM can correct the problems of individual heterogeneity, missing variable deviation, measurement error, and potential endogeneity that are not observed. These problems often affect the estimation effect of the model when using pool OLS and a static panel model. In addition, the system GMM method can reduce the potential bias and inaccuracy caused by the estimation method. 

In the static panel model, the fixed effect model and the random effect model are the most common. In this study, the static panel model is used to test the stability. The linear model is set as follows:

(9) $$Y_{it}=\alpha_{i}+\lambda_{t}+X_{it}\beta +\epsilon_{it}$$

where $\alpha_{i}$ is the individual effect, which means those factors that do not change with time, and $\lambda_{t}$ is the time effect, which is used to control the influence of the factors that change with time. However, in most cases, $\alpha_{i}$ and $\lambda_{t}$ cannot be directly observed or quantified, thus, they cannot enter the model. The panel data model can be divided into the fixed effect model and random effect model. Taking the individual dimension as an example, when $\alpha_{i}$ and $X_{it}$ are correlated (i.e.$,\;\mathrm{corr}\left(\alpha_{i},X_{it}\right)\neq 0$), the model is a fixed effect model; otherwise, it is a random effect model [39]. In other words, given i, if $\alpha_{i}$ is a certain value, it is a fixed effect model (different individuals have different characteristics, and each individual has a special nominal value). Otherwise, it is a random effect model (the difference of different individuals belongs to a random phenomenon and obeys a normal distribution). Similarly, there is a distinction between the fixed effect and random effect for the time dimension t and $\lambda_{t}$. The difference between the two models is mainly reflected in the treatment of the “individual effect”. The fixed effect model assumes that the individual effect is fixed in the group, and the differences between individuals are reflected in each individual having a specific intercept term, whereas the random effect model assumes that all individuals have the same intercept term, and the differences between individuals are random, and these are mainly reflected in the setting of the random interference term. The choice of a fixed effect model and random effect model can be judged by the Hausmann test.

We take ESTFP as the explained variable, and its data is estimated by the previous DEAP 2.1 software. It should be noted that the data is the growth index of the current year relative to the previous year, and it cannot be used as the explanatory variable directly. The treatment of this study is based on the first year’s ESTFP, which is multiplied to every subsequent year, so that the current year’s ESTFP value can be obtained and included in the model for empirical research. The most important explanatory variable is the ecological index (EI). The other control variables are the number of employees at the end of the year (labor), total imports and exports (open), and the local general public budget expenditure (epd). The specific setting of the indicators is shown in Table 2 below. According to Managi and Jena [40], and Zhou and Zheng [41], this study converts all variables into (1 + variables), and then conducts logarithmic processing. 

In order to find out whether there is inertia in the change of the ESTFP, this study establishes the following dynamic panel regression model, in which $lnestfp_{i,t-1}$ represents the first lag term of the change rate of ESTFP:

(10) $${\mathrm{lnESTFP}}_{i,t}=\beta_{0}+\beta_{1}{\mathrm{lnEI}}_{i,t}+\beta_{2}{\mathrm{lnabor}}_{i,t}+\beta_{3}{\mathrm{lnopen}}_{i,t}+\beta_{4}{\mathrm{lnepd}}_{i,t}+\beta_{5}{\mathrm{lnESTFP}}_{i,t-1}+\varepsilon_{i,t}.\;$$

where $\beta_{0}$ is the constant term, $\varepsilon_{i,t}$ is the residual term, and subscripts i and t denote individual and time, respectively. $\beta_{1},\beta_{2},\beta_{3},\beta_{4},{\mathrm{and}\;\beta }_{5}$ represent the coefficient term of the corresponding regression variable. Table 2 shows the symbol expression, meaning, measurement method, symbol expectation, and data source of the core explanatory variables and control variables. Table 3 provides the basic statistical description of each variable.

### 2.4. Research Hypothesis

**Figure 5:**
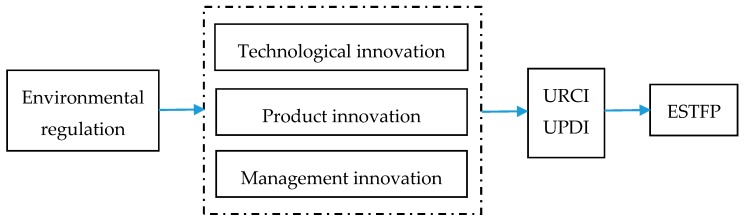
Channels of Environmental Regulations Affecting ESTFP.

Under various environmental regulations, enterprises are bound to face the rising costs of pollution discharge and production, and the operating profit of enterprises is difficult to maintain under the pressure of high compliance costs. Based on the assumption of the “rational economic man”, this will inevitably force enterprises to carry out a series of innovations and reforms to improve their viability and competitiveness. These will include technological innovation, product innovation, management innovation, and other initiatives, as shown in Figure 5.

**Hypothesis** **1.**  *Based on the environmental regulations, ESTFP, with the urban resource consumption index (URCI) and urban pollution discharge index (UPDI), is more efficient than traditional market TFP*.

**Figure 6:**
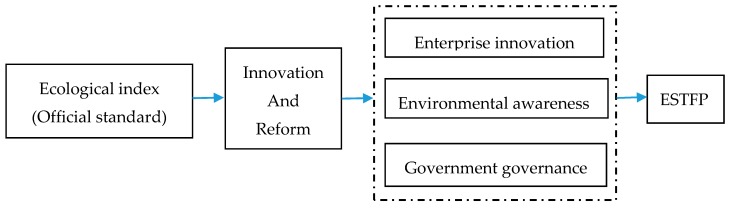
Channels of Influence of the Ecological Index on ESTFP.

Based on the official standards issued by the Ministry of Environmental Protection, governments at all levels continue to stimulate local enterprises to carry out technological innovation, improve the unit output of enterprise products, and reduce the unit resource consumption and unit pollution discharge of enterprise products. At the same time, they will continue to guide the public to increase their awareness of environmental protection and reduce their consumption of individual resources in order to improve the ecological environment of the whole city, as shown in Figure 6. According to the EI data in Table 1, the EI has experienced the process of declining first and then rising, which indicates that in recent years the ecological environment of most prefecture-level cities in Guangdong Province has improved, and the ESTFP should be improved.

**Hypothesis** **2.**  *There is a positive correlation between the EI and ESTFP*.

## 3. Results and Discussion

### 3.1. Estimations of ESTFP and Verification of Research Hypotheses 

#### 3.1.1. ESTFP Results and Discussion

Based on the input-oriented measures, this study uses DEAP 2.1 software to calculate the trend and decomposition results of the ecological sustainable total factor productivity change (ESTFPC) in 21 prefecture-level cities of Guangdong Province in 2008–2016. The specific data are shown in Table 4.

According to Table 4, the biggest improvement of ESTFPC is in Shanwei, which has reached 12.5%, and the biggest decline is in Chaozhou, which has reached −16%. The differences in the ESTFPC among the cities is large, which shows that the technical improvement of the 21 prefecture-level cities in Guangdong is not balanced, and the differences between the regions are large. There are six cities that have improved the three indexes of ESTFPC, ESTC, and ESPEC, and they account for 28.57%. There are seven cities that have improved the two indexes of ESEC and ESSEC, and they account for 33.33% of the total. For the 21 prefecture-level cities in Guangdong Province as a whole, the mode of economic growth still needs to be further improved and adjusted. For the cities with low ESTFPC, this means that the improvement of technological progress and efficiency change has not formed a general atmosphere, thus, the economic development path of the 21 prefecture-level cities in Guangdong Province faces numerous opportunities and challenges.

The decomposition results of ESTFPC show that the growth of ESTFPC in the 21 prefecture level cities of Guangdong Province is mainly caused by ESTC, because the six cities with ESTFPC growth are also the six cities with ESTC growth. This also shows that ESTC plays a leading role in ESTFPC. Conversely, in the 15 cities where ESTFPC declines, their ESTC also decreases correspondingly. The low eco-sustainable technology change (ESTC), that is, the low technology efficiency, reflects the fact that the existing technology in most cities of Guangdong has not been fully and effectively utilized, which is also consistent with the drag of the technology efficiency change on TFP proposed by Liu and Li [42]. The main reason is the transformation of the economic structure and the change of the industrial structure [43].

#### 3.1.2. Research Hypothesis Verification on ESTFP

In order to verify the previous research hypothesis, we exclude the two dimensions of URCI and UPDI and only use the traditional TFP framework to measure and compare the results. See Table 5 for the results.

From Table 5 it can be seen that the results calculated by integrating the URCI and UPDI into the TFP framework are better than those calculated by the traditional TFP framework. From the average, except that ESSEC and SEC are equal, the other efficiency changes under the framework of ecological sustainability are better than those under the traditional framework. For example, the ESTFPC is 0.96, which is higher than the traditional TFPC of 0.954. Appropriate environmental regulations can promote enterprises to carry out more innovation activities. When the profits brought by innovation can make up for or even exceed the compliance costs, enterprises can realize the double promotion of economic and environmental benefits, which is consistent with the Porter hypothesis [44]. It is also similar to the research results of the Chinese scholars Wang et al. [45]. This also verifies the hypothesis that the first one is correct; that is, based on environmental regulations, the ESTFP with URCI and UPDI is more efficient than the traditional market TFP.

### 3.2. Empirical Research Results and Hypothesis Verification of the Relationship between the Ecological Environment and ESTFP

#### 3.2.1. Analysis of Regression Results of the Dynamic Panel Model

In order to avoid the multicollinearity problem, we need to test the variance inflation factor of the variables (VIF) so we can screen out the better independent factors to enhance the explanatory ability of the model. The VIF is a method to judge whether there is multicollinearity by examining the degree to which a given explanatory variable is interpreted by all the other explanatory variables in the equation. Every explanatory variable in the equation has a VIF, which reflects the index of how much multicollinearity increases the variance of the estimation coefficient. The formula of the VIF is ${\mathrm{VIF}}_{k}=\frac{1}{1-R_{k}^{2}}$. $R_{k}^{2}$ is the determinable coefficient of the multiple explanatory variables. The larger $R_{k}^{2}$ is, the more serious the multicollinearity is, and the larger ${\mathrm{VIF}}_{k}$ is. Experience shows that there is a serious multicollinearity between the explanatory variables and other explanatory variables when ${\mathrm{VIF}}_{k}\geq 10$.

Before the panel regression, the VIF is used to test whether there is multicollinearity among the variables. The test result is that the VIF of each variable is less than 10, and the average value is 1.47, thus, there is no multicollinearity problem among the variables of the model. 

In this study the system GMM method in Stata 15.0 was used to estimate the model, and the Sargan test and Arellano-Bond (AR2) test were used to verify the existence of over-identification and disturbance items. The original hypothesis of the Sargan test is that “all instrument variables are valid”. If the *p* value is greater than 10%, it means the original hypothesis cannot be rejected at the 10% significant level, and thus the selection of the instrument variables is appropriate. The AR(2) is a test for the second-order autocorrelation of the difference of the disturbance term. The system GMM estimates that the *p* value through AR(2) needs to be greater than 10%, and it is better when the AR(2) *p* value is bigger. The test results are shown in Table 4. First, the Sargan test *p* values of models (1)–(4) were all above 0.1, which meant that the instrument variables were reasonable as a whole, and the original assumption that the instrument variables were not over-recognized was accepted. Second, the *p* values of AR(2) were greater than 0.1, which indicated that the GMM estimators of the system were consistent, and there was no second-order autocorrelation in the model. Therefore, the model setting was reasonable, and the estimation results had strong reliability.

It can be seen from Table 6, if we take model (4) as an example, that the stepwise regression results of each explanatory variable have good consistency. This study focuses on the EI and ESTFP at the level of a 1% significantly positive correlation (β = 0.725, *p* < 0.01), which indicates that the ecological environment has a significant role in promoting urban production efficiency; the better the environment, the higher the urban efficiency. In addition, it was found that the first lag coefficient of ESTFP is positive, and it has a significant promoting effect on the current period of ESTFP at the level of 1% (β = 0.602, *p* < 0.01). This shows that the change of a city’s ESTFP is greatly influenced by the change of its last period, and there is a strong inertia of the city’s ESTFP itself. The regression results also show that the total import and export volume (open), which reflects the degree of opening-up, is significantly negative correlated with ESTFP at the level of 1% (β = −0.019, *p* < 0.01). This conclusion is also supported by many scholars [46,47]. They believe that in the process of trade liberalization, countries will reduce their environmental quality standards to maintain or enhance their trade competitiveness. Therefore, import and export trade is an important factor in the aggravation of environmental pollution; that is, import and export trade is negatively related to ESTFP. In addition, they also believe that FDI, which belongs to the category of opening-up to the outside world, has the same inhibitory effect on TFP. In the case where the ecological environment is not damaged, the healthy development of foreign trade should take into account the total growth and quality improvement of foreign trade. All regions should pursue a good balance between the harmonious development of the import and export trade and the ecological environment. The number of workers at the end of the year (labor) is significantly positively correlated with ESTFP at the level of 10% (β = 2.193, *p* < 0.1). This conclusion is the same as in many research results. Since Schulz’s human capital theory was put forward, human capital has been regarded as an important variable that causes economic growth. Non-Chinese scholars have come to the conclusion that human capital contributes to the improvement of TFP from both the theoretical and empirical aspects [48]. For the first time, Yue and Liu used human capital as endogenous factor to measure TFP and found that the level of TFP at the provincial level in China was significantly underestimated when it was not included in human capital [49]. In recent studies human capital has been regarded as an exogenous variable, and the mechanism of its action on TFP has been investigated. The results show that the improvement of human capital stock and its allocation efficiency can significantly improve TFP, but the effect of human capital on TFP at different levels is different [50]. 

#### 3.2.2. Stability Test

The regression results of the random effect model and the fixed effect model are reported in Table 7. Finally, the Hausman test is used to select the two models, and the test results show that the original hypothesis of using the random effect model cannot be rejected. Therefore, this study should choose the random effect model.

According to Table 7, with the increase of the explanatory variables, the Adj *R^2^* gradually increases to 0.7298, and the model has a high explanatory power for ESTFP. Next, we take model (4) as an example to analyze the influence of each variable on the difference of ESTFP. According to the regression results, we can get the following conclusions. The two explanatory variables of “EI” and “labor” have a significant role in promoting ESTFP; that is, with the improvement in the ecological environment and the increase of the labor force, its ESTFP can be improved. The variables “open” and “epd” have significant negative effects on ESTFP; that is, with the increase of the import and export trade and the budget expenditure, ESTFP will decline. The above conclusion is consistent with that of the system GMM, and the conclusion is stable.

#### 3.2.3. Discussion on Regression Results of Panel Data and Verification of Research Hypotheses

In this study the system GMM model, the random effect model, and the fixed effect model have been used to empirically study the relationship between the ecological environment and ESTFP. The comprehensive results are shown in Table 8.

The GMM regression results show that the core explanatory variable “ecological index (EI)” is significantly positively correlated with ESTFP at the level of 1%. The main reasons are as follows. First, government governance. As the government has the primary role in environmental protection, the relevant laws and regulations, policy documents, and industry standards issued by the government all play a leading, guiding, supervising, and punishing role in the whole ecological environment in an endeavor to ensure that the relevant production enterprises act according to the rules, produce according to the standards, and have standards to find. Second, technological innovation. Enterprises are important participants in the whole ecological environment; their every action has a relationship to the quality of the ecological environment. For the sake of high profits, enterprises ignore environmental protection and thereby earn “toxic profits”, but this behavior may also be punished by the government. Based on this background, at present a large number of Chinese production enterprises are carrying out technological innovation, improving the efficiency of their production links, and improving the standard treatment of harmful substances such as sewage, exhaust gas, smoke, and dust. Third, environmental awareness. At present, the awareness of environmental protection of the whole society has gradually increased, and it has reached an unprecedented level. The opening of environmental protection courses in primary and secondary schools, and the garbage classification vigorously advocated by the government departments, are all good demonstration cases. From this point of view, the ecological environment of the whole society is bound to improve and promote the improvement of ESTFP. Therefore, this also verifies the hypothesis that the ecological environment has a positive role in promoting ESTFP, and EI has a positive correlation with ESTFP. This conclusion is also similar to that of Zhu and Wang [51], who believed that environmental regulation has a positive impact on green total factor productivity and green technology progress. Reasonable environmental regulations urge enterprises to internalize the external cost of environmental regulation and encourage enterprises to carry out technological innovation activities in order to improve the input-output level, which can partially or completely offset the cost rise caused by environmental regulation, increase the net income, and produce an “innovation compensation effect”.

There is a significant positive correlation between the labor and ESTFP at the level of 10%. The increase of the labor force will bring positive economic development, while the promotion of human capital will make the economic quality and economic efficiency higher on the basis of this economic development. The main reasons are as follows. First, the improvement of human capital involves the improvement of the intelligence and professional skills of ordinary workers, which will undoubtedly improve the productivity of workers. Second, Guangdong Province is one of the most active provinces in China. Every year the surplus labor force from all over the country flows to Guangdong Province. They bring advanced ideas, technology, and frontier knowledge, which has a positive role in promoting ESTFP. This is also similar to the views of Kim and Park [52] and Balcerzak and Pietrzak [53]. Based on the above conclusions, we suggest that the relevant functional departments should establish an independent innovation talent system, which involves actively building various training, cooperation, and exchange platforms and providing continuous education opportunities for employees to improve their skills, so they can further improve their work efficiency. We should adopt a flexible salary system and reward measures for employees, abolish the old rules and regulations that are contrary to incentivizing innovation, and cut down the red tape that hinders the release of innovation vitality.

There was a significant negative correlation between the total import and export (open) and ESTFP at the 1% level. As the largest import and export province in China, Guangdong has a large scale of imports and exports, but its efficiency has not been improved. This shows that the transformation rate of a large number of import and export enterprises in Guangdong, in terms of progressing their ecological sustainable technology and their ability to effectively use existing resources, need to be improved, which is also in line with the view of Yang and Han [54]. Based on this, the current large-scale trade transactions, which are at the cost of high energy consumption and high pollution, cannot continuously promote the improvement of ecologically sustainable total factor productivity. Improving the quality and utilization of foreign investment is an effective way to avoid low efficiency. The specific measures are as follows. First, improve the environmental threshold of the import and export trade enterprises. Firmly resist the import and export enterprises with “high investment, high consumption, high pollution, and low efficiency”. Second, actively guide import and export enterprises in Guangdong towards ecological sustainability. Encourage import and export enterprises in Guangdong to invest in foreign exchange earning products that are high-tech products and environmental protection products.

## 4. Conclusions

Based on the literature review and research hypothesis, this study draws the following three main conclusions. First, according to the EI data, in the past 10 years the ecological environment of the 21 prefecture-level cities in Guangdong Province has generally declined first and then increased, thus, the ecological environment has changed from good to bad and then from bad to good. Second, through the empirical test using the DEA Malmquist model, it was found that the ESTFP with URCI and UPDI is more efficient than the traditional market TFP. Accordingly, hypothesis 1 is correct. Third, in this study the GMM model of the dynamic panel system is used for the empirical study, and the random effect model and fixed effect model of the static panel are used to test its stability. The results show that the ecological environment has a significant positive role in promoting ESTFP at the level of 1%. Accordingly, hypothesis 2 is correct.

The contributions of this study are as follows. First, this study proposes a new index called ecological sustainable total factor productivity (ESTFP). This can better measure a city’s economic development while taking ecological sustainability into account by adding the two dimensions of urban resource consumption and urban pollution discharge into the traditional TFP framework. Second, we use the DEA-Malmquist method to measure the changes and components of ESTFP in 21 prefecture level cities of Guangdong Province from 2009 to 2016. Third, this may be the first empirical study on the relationship between the ecological environment and the ESTFP.

The possible limitations of this study as fellows. First that the research object is limited to only one province of China, thus, the national and international implications may be different and the conclusions of this study may be different with an increase in the number of samples. Therefore, in order to further extend this study, it is suggested that research should be conducted in more cities, and preferably be nationwide. Second, due to the unavailability of data, some important influencing factors, such as the quantified data of environmental awareness, as well as policies and regulations, could not be incorporated into the analyses of the relative ecological process in this study. Analyses of those factors may be possible in future research work when the relative data are available.

## Figures and Tables

**Table 1:** Ecological Index (EI) of Guangdong Province in 2008–2017.

City	2008	2009	2010	2011	2012	2013	2014	2015	2016	2017
Guangzhou	76	75	63	61	62	63	62	63	64	62
Shaoguan	89	87	78	77	79	79	82	83	84	85
Shenzhen	83	76	74	73	72	73	65	66	67	69
Zhuhai	100	76	75	72	73	73	70	69	69	71
Shantou	73	73	66	66	67	68	66	66	68	67
Foshan	61	60	58	56	58	59	62	63	63	61
Jiangmen	86	86	72	69	72	72	75	74	75	77
Zhanjiang	68	69	63	63	64	64	65	64	66	67
Maoming	79	77	68	66	68	70	72	72	75	78
Zhaoqing	85	81	73	72	74	74	80	80	80	82
Huizhou	89	83	74	75	76	78	81	81	83	81
Meizhou	84	80	75	73	74	77	79	80	83	84
Shanwei	90	84	75	74	75	78	77	77	79	80
Heyuan	90	87	78	76	77	78	80	81	83	83
Yangjiang	86	88	76	74	76	76	77	77	78	82
Qingyuan	85	82	76	74	76	78	81	82	83	84
Dongguan	61	61	61	58	60	61	60	60	62	60
Zhongshan	85	72	68	65	67	68	67	66	67	64
Chaozhou	76	73	68	66	67	69	70	71	74	77
Jieyang	81	77	70	68	69	72	71	71	74	74
Yunfu	73	70	67	65	67	67	73	72	74	82
mean	80.95	77	70.38	68.71	70.14	71.29	72.14	72.29	73.86	74.76

Data source: Department of Ecology and Environment of Guangdong Province.

**Table 2:** Indicators of Influencing Factors of ESTFP.

Influence Factor	Indicators	Measurement Method	Symbol Anticipation	Data Sources
environmental effect	Ecological index (EI)	See Formula (1)	+	Guangdong Statistics Yearbooks
Opening to the outside world	Total imports and exports (open)	total imports and exports/GDP	+	
human capital	Number of employees in each city at the end of the year (labor)	Original data is not adjusted	+	
government intervention	Fiscal expenditure (epd)	Local general public budget expenditure/GDP	+	

**Table 3:** Descriptive Statistics of Variables.

Variables	Mean	SD	Min	Max	N
lnESTFP	0.579	0.177	0.221	1.271	168
lnEI	4.285	0.097	4.043	4.489	168
lnopen	0.830	0.654	0.033	2.696	168
lnlabor	0.028	0.020	0.009	0.089	168
lnepd	0.130	0.050	0.059	0.335	168

**Table 4:** Efficiency Change and Decomposition.

City	ESEC	ESTC	ESPEC	ESSEC	ESTFPC
Guangzhou	1	1.062	1	1	1.062
Shenzhen	1	0.984	1	1	0.984
Zhuhai	1	0.924	1	1	0.924
Shantou	0.964	0.909	0.96	1.004	0.876
Foshan	1	1.024	1	1	1.023
Shaoguan	1	0.96	1	1	0.96
Heyuan	0.973	0.91	0.961	1.013	0.886
Meizhou	1	0.863	1	1	0.863
Huizhou	1.007	0.985	1.009	0.998	0.992
Shanwei	1.037	1.085	1	1.037	1.125
Dongguan	1.017	0.9	1.014	1.003	0.916
Zhongshan	0.998	0.957	1.01	0.987	0.955
Jiangmen	0.966	0.951	0.98	0.986	0.919
Yangjiang	1.041	0.885	1.022	1.018	0.921
Zhanjiang	1.049	1.027	1.055	0.994	1.077
Maoming	1	0.962	1	1	0.962
Zhaoqing	1.014	0.984	1.014	1	0.998
Qingyuan	0.967	1.035	0.975	0.992	1.001
Chaozhou	1.012	0.83	1	1.012	0.84
Jieyang	0.96	1.06	0.95	1.01	1.018
Yunfu	1	0.918	1	1	0.918
mean	1	0.96	0.997	1.003	0.96

**Table 5:** Comparison between ESTFP Framework and Traditional TFP Framework.

Year	ESTFP Framework					Traditional TFP Framework				
	ESEC	ESTC	ESPEC	ESSEC	ESTFPC	EC	TC	PEC	SEC	TFPC
2008–2009	1.039	0.933	1.021	1.017	0.969	0.998	0.859	1	0.998	0.858
2009–2010	1.017	0.852	1.023	0.994	0.867	0.981	0.944	0.983	0.997	0.926
2010–2011	1.029	0.896	1.016	1.013	0.921	1.01	0.959	1.003	1.007	0.968
2011–2012	0.995	0.954	0.98	1.015	0.949	1.005	0.957	1.009	0.996	0.962
2012–2013	0.975	0.938	0.969	1.007	0.915	1.047	0.939	1.022	1.024	0.983
2013–2014	0.978	1.016	0.987	0.991	0.994	0.975	0.992	0.996	0.979	0.968
2014–2015	0.988	0.976	0.983	1.005	0.964	0.982	1.004	0.97	1.013	0.985
2015–2016	0.981	1.144	1.001	0.981	1.123	0.992	0.996	0.98	1.012	0.988
mean	1	0.96	0.997	1.003	0.960	0.998	0.955	0.995	1.003	0.954

**Table 6:** Regression Results of the System GMM Model.

Indicators	(1)	(2)	(3)	(4)
	LnESTFP	LnESTFP	LnESTFP	LnESTFP
L.LnESTFP	0.639 ***	0.656 ***	0.601 ***	0.602 ***
	(28.16)	(26.78)	(23.28)	(20.48)
LnEI	0.635 ***	0.655 ***	0.771 ***	0.725 ***
	(6.92)	(8.18)	(12.52)	(9.08)
Lnlabor		−0.660 *	1.044 ***	2.193 *
		(−1.74)	(3.55)	(1.82)
Lnopen			−0.020 ***	−0.019 ***
			(−12.58)	(−6.02)
Lnepd				0.020
				(0.33)
_cons	−2.528 ***	−2.607 ***	−3.102 ***	−2.934 ***
	(−6.3)	(−7.29)	(−11.71)	(−9.02)
*N*	147	147	147	147
AR(1)-*p*	0.0388	0.0389	0.0355	0.0361
AR(2)-*p*	0.1364	0.1364	0.1321	0.1319
Sargan-*p*	0.4306	0.4097	0.4479	0.4568

Note: the value in parentheses is the Z statistic; * *p* < 0.1, ** *p* < 0.05, *** *p* < 0.01.

**Table 7:** Regression Results of Random Effect Model and Fixed Effect Model.

Indicators	Random Effect Model				Fixed Effect Model			
	(1)	(2)	(3)	(4)	(1)	(2)	(3)	(4)
	LnESTFP	LnESTFP	LnESTFP	LnESTFP	LnESTFP	LnESTFP	LnESTFP	LnESTFP
LnEI	0.140 (0.84)	0.204 (1.18)	0.303 * (1.84)	0.378 ** (2.32)	0.237 (1.29)	0.237 (1.27)	0.352 ** (1.99)	0.400 ** (2.31)
Lnlabor		2.138 (1.36)	4.567 *** (2.76)	3.887 ** (2.32)		0.020 (0.01)	6.507 ** (2.00)	6.226 * (1.95)
Lnopen			−0.079 ***	−0.052 ***			−0.085 ***	−0.057 ***
			(−4.55)	(−2.74)			(−4.51)	(−2.70)
Lnepd				−0.884 ***				−0.908 ***
				(−2.93)				(−2.77)
_cons	−0.023	−0.356	−0.782	−0.993	−0.437	−0.438	−1.041	−1.148
	(−0.03)	(−0.47)	(−1.09)	(−1.40)	(−0.56)	(−0.54)	(−1.34)	(−1.51)
*N*	168	168	168	168	168	168	168	168
Within *R^2^*	0.0113	0.0078	0.1317	0.1747	0.0113	0.2638	0.1335	0.1777
Adj R^2^	0.6818	0.6796	0.7172	0.7298	0.6818	0.6796	0.7172	0.7298

Note: For the random effect models, the value in parentheses is the Z statistic; for the fixed effect models, the value in parentheses is the T statistic; * *p* < 0.1, ** *p* < 0.05, *** *p* < 0.01.

**Table 8:** Comprehensive Summary of Regression Results.

Indicators	(1)	(2)	(3)		(1)	(2)	(3)
	FE	RE	SYSGMM		FE	RE	SYSGMM
LnEI	0.400 **	0.378 **	0.725 ***	L.lnTFP			0.602 ***
	(2.31)	(2.32)	(9.08)				(20.48)
Lnlabor	6.226 *	3.887 **	2.193 *	_cons	−1.148	−0.993	−2.934 ***
	(1.95)	(2.32)	(1.82)		(−1.51)	(−1.40)	(−9.02)
Lnopen	−0.057 ***	−0.052 ***	−0.019 ***	Within *R^2^*	0.1777	0.1747	
	(−2.70)	(−2.74)	(−6.02)	Adj. *R^2^*	0.7298	0.7298	
Lnepd	−0.908 ***	−0.884 ***	0.020	AR(2)-*p*			0.1319
	(−2.77)	(−2.93)	(0.33)	Sargan-*p*			0.4568

Note: For the random effect model and system GMM model, the value in parentheses is the Z statistic; for the fixed effect model, the value in parentheses is the T statistic; * *p* < 0.1, ** *p* < 0.05, *** *p* < 0.01.

## Appendix A

1. Biological richness index = (BI + HQ)/2
  where BI is the biodiversity index and HQ is the habitat quality index.
2. Vegetation coverage index= NDVI_Regional mean_=$A_{\mathrm{veg}}\times \left(\frac{\sum_{i=1}^{n}P_{i}}{n}\right)$
  In the formula, Pi is the average value of the maximum monthly NDVI value of the image elements from May to September, and it is recommended to use the NDVI data of MOD13 with a spatial distribution rate of 250 m. Variable n is the number of regional image elements, and Aveg is the normalization index of the vegetation coverage index with a reference value of 0.0121165124.
3. Water network denseness index = $\left(A_{\mathrm{riv}}\times \frac{River\;length}{\mathrm{Area}}+A_{\mathrm{lak}}\times \frac{\mathrm{Water}\;\mathrm{area}\;\left(\mathrm{Lake},\;\mathrm{reservoir},\;\mathrm{channel},\;\mathrm{offshore}\right)}{\mathrm{Area}}+A_{\mathrm{res}}\times \frac{\mathrm{Water}\;\mathrm{resources}}{\mathrm{Area}}\right)/3$
  where $A_{\mathrm{riv}}$ is the normalized index of the river length, and the reference value is 84.3704083981; $A_{\mathrm{lak}}$ is the normalized index of the water area, and the reference value is 591.7908642005; $A_{\mathrm{res}}$ is the normalized index of the water resources, and the reference value is 86.3869548281.
4. Land stress index = $A_{\mathrm{ero}}\times \left(0.4\times \mathrm{Heavy}\;\mathrm{erosion}\;\mathrm{area}+0.2\times \mathrm{Moderate}\;\mathrm{erosion}\;\mathrm{area}+0.2\times \mathrm{Construction}\;\mathrm{land}\;\mathrm{area}+0.2\times \mathrm{Other}\;\mathrm{land}\;\mathrm{stress}\right)$/Area
  In the formula, $A_{\mathrm{ero}}$ is the normalized index of the land stress index, and the reference value is 236.0435677948.
5. $\begin{array}{ll} \mathrm{Pollution}\;\mathrm{load}\;\mathrm{index}= & 0.2\times A_{\mathrm{COD}}\times \frac{\mathrm{COD}\;\mathrm{Emissions}}{\mathrm{regional}\;\mathrm{annual}\;\mathrm{precipitation}}\;+\\ & 0.2\times A_{NH3}\times \frac{\mathrm{Nitrogen}\;\mathrm{and}\;\mathrm{oxygen}\;\mathrm{emissions}}{\mathrm{Regional}\;\mathrm{annual}\;\mathrm{precipitation}}\;\;+\\ & 0.2\times A_{\mathrm{SO}2}\times \frac{{\mathrm{SO}}_{2}\mathrm{emissions}}{\mathrm{Area}}\;+\\ & 0.1\times A_{\mathrm{YFC}}\times \frac{\mathrm{Smoke}\;\left(\mathrm{powder}\right)\;\mathrm{dust}\;\mathrm{emission}}{\mathrm{Area}}\;+\\ & 0.2\times A_{\mathrm{NOX}}\times \frac{\mathrm{NOX}\;\mathrm{emissions}}{\mathrm{Area}}\;+\\ & 0.1\times A_{\mathrm{SOL}}\times \frac{\mathrm{Solid}\;\mathrm{waste}\;\mathrm{disposal}}{\mathrm{Area}} \end{array}$
  In the formula, $A_{\mathrm{COD}}$ is the normalization coefficient of COD, and the reference value is 4.3937397289; $A_{NH3}$ is the normalization coefficient of ammonia nitrogen, and the reference value is 40.1764754986; $A_{\mathrm{SO}2}$ is the normalization coefficient of ${\mathrm{SO}}_{2}$, and the reference value is 0.0648660287; $A_{\mathrm{YFC}}$ is the normalization coefficient of smoke (powder) dust, and the reference value is 4.0904459321; $A_{\mathrm{NOX}}$ is the normalization coefficient of nitrogen oxides, with a reference value of 0.5103049278; $A_{\mathrm{SOL}}$ is the normalization coefficient of solid waste, and the reference value is 0.0749894283.
6. Environmental limit index
  The environmental restriction index is a restrictive index of the ecological environment. It refers to the restriction and adjustment of the ecological environment according to the ecological damage and environmental pollution in the region.
